# Toxic Metals in Cereals in Cape Verde: Risk Assessment Evaluation

**DOI:** 10.3390/ijerph18073833

**Published:** 2021-04-06

**Authors:** Carmen Rubio-Armendáriz, Soraya Paz, Ángel J. Gutiérrez, Verena Gomes Furtado, Dailos González-Weller, Consuelo Revert, Arturo Hardisson

**Affiliations:** 1 Department of Toxicology, Universidad de La Laguna, 38071 La Laguna (Canary Islands), Spain; spazmont@ull.edu.es (S.P.); ajguti@ull.edu.es (Á.J.G.); dgonwel@gmail.com (D.G.-W.); atorre@ull.edu.es (A.H.); 2 Entidade Regulatora Independiente da Saúde, Avenida Cidade de Lisboa, 296-A Praia, Cape Verde; Verena.Furtado@eris.cv; 3 Health Inspection and Laboratory Service, Servicio Canario de Salud, 38004 S/C de Tenerife (Canary Islands), Spain; 4 Departament of Physical Medicine and Pharmacology, Universidad de La Laguna, 38071 La Laguna (Canary Islands), Spain; mgirones@ull.edu.es

**Keywords:** Cape Verde, cereals, metals, dietary intake, risk assessment

## Abstract

Consumption of cereals and cereal-based products represents 47% of the total food energy intake in Cape Verde. However, cereals also contribute to dietary exposure to metals that may pose a risk. Strengthening food security and providing nutritional information is a high-priority challenge for the Cape Verde government. In this study, toxic metal content (Cr, Ni, Sr, Al, Cd, and Pb) is determined in 126 samples of cereals and derivatives (rice, corn, wheat, corn flour, wheat flour, corn gofio) consumed in Cape Verde. Wheat flour samples stand out, with the highest Sr (1.60 mg/kg), Ni (0.25 mg/kg) and Cr (0.13 mg/kg) levels. While the consumption of 100 g/day of wheat would contribute to 13.2% of the tolerable daily intake (TDI) of Ni, a consumption of 100 g/day of wheat flour would contribute to 8.18% of the tolerable weekly intake (TWI) of Cd. Results show relevant Al levels (1.17–13.4 mg/kg), with the highest level observed in corn gofio. The mean Pb average content in cereals is 0.03–0.08 mg/kg, with the highest level observed in corn gofio. Al and Pb levels are lower in cereals without husks. Without being a health risk, the consumption of 100 g/day of wheat contributes to 17.5% of the European benchmark doses lower confidence limit (BMDL) of Pb for nephrotoxic effects; the consumption of 100 g/day of corn gofio provides an intake of 1.34 mg Al/day (13.7% of the TWI) and 8 µg Pb/day (20% of the BMDL for nephrotoxic effects). A strategy to minimize the dietary exposure of the Cape Verdean population to toxic metals from cereals should consider the continuous monitoring of imported cereals on arrival in Cape Verde, the assessment of the population’s total diet exposure to toxic metals and educational campaigns.

## 1. Introduction

The Macaronesian region consists of a collection of four volcanic archipelagos in the North Atlantic Ocean (Cape Verde, Azores and Madeira in Portugal and the Canaries in Spain). The four archipelagos share features such as a volcanic origin, a contrasting landscape, a gentle climate and a particularly rich biodiversity. The archipelago of Cape Verde is located on the West African coast, 500 km from Senegal, and comprises ten islands, nine of which are inhabited and one of which is uninhabited. The population of the island of Santiago is approximately 260,000 inhabitants, while that of São Vicente is 76,000. The Cape Verdean diet is characterized by the consumption of significant amounts of cereals and cereal-based products. According to the preliminary results of the 2015 Ínquérito Ás Despesase e Receitas Familiares (IDRF), the ingestion of cereals occupies the highest annual per capita consumption expenditure (about 11,611$) compared to other food products consumed. However, internal cereal production satisfies only 6.9% of the population’s consumption needs, contributing to the highly vulnerable state of the country regarding food security. Food security in Cape Verde is also affected by agroclimatic variations and external market fluctuations. National cereal production in 2019 was estimated at about 1000 tons, almost 70% below the mean average of the previous five years [1]. Therefore, about 85% of the domestic cereal demand (mostly rice and wheat for human consumption) was covered by imports. The cereal import requirements in the 2019/2020 marketing year (November to October) were forecasted at an above‑average level of 87,000 tons [1]. From 2016 to 2020, the cereal imports reached a total of 419,749.30 tons, with an emphasis on corn (159,979.30 tons), rice (144,799.33 tons) and wheat grain (91,623.39 tons). The market supply of cereals stems both from food aid through cooperative relations with development partners and through commercial imports [2]. Current domestic corn production does not meet the internal demand, and so the cereal must be imported for food and fodder [3]. Moreover, the main drivers of food insecurity in Cape Verde are the effects of dry weather events (such as drought) and pest attacks on cereal and fodder production [1]. As mentioned above, food insecurity in Cape Verde has a structural and multifactorial nature: It demonstrates a structural deficit in national food production, strong dependence on the international market and economic accessibility weaknesses. Strengthening the Food Security and Nutrition Information System (FSNIS) is an important challenge for the Cape Verde government [4].

According to the Food and Agriculture Organization (FAO), in 2017, about 13% of the population was undernourished. The data available indicate that 20% of rural families lived in a situation of food insecurity, with 13% in a moderate position and 7% in a severe position [2]. Cape Verde is in a nutritional transition period characterized both by the high consumption of fat, refined carbohydrates, cholesterol and sugar, and by the low consumption of fruit and vegetables, causing a rapid and significant increase in the prevalence of being overweight and obese [5]. However, the consumption of cereals and cereal-based products is still relevant, representing 47% of the total food energy intake. In Cape Verde, the cereal balance for 2002/2003 estimated a cereal consumption of 242 kg/year per person, comprising 123 kg of corn (337 g/day), 67 kg of rice (184 g/day) and 52 kg of wheat (142 g/day).

Although the nutritional value of cereals is noteworthy, cereals may also contain elements that are harmful to health [6,7], as is the case with elements such as Al, Cd, Cr, Ni, Pb and Sr. Each of these elements has standards of tolerable daily/weekly intake (TDI/TWI) and/or benchmark dose (lower confidence limit) (BMDL) levels set by reference bodies in food safety, such as the European Food Safety Authority (EFSA) and the World Health Organization (WHO) (Table 1).

Al is a neurotoxic metal with no function in the human body [14]. Prolonged exposure to Al is related to neurodegenerative diseases such as Alzheimer*’*s, and the estimation of its dietary exposure is the subject of previous studies [15,16,17]. In 2008, the EFSA estimated the dietary intake of Al in the European population to be 0.2–1.5 mg/kg of body weight per week for an adult weighing 60 kg, and concluded that cereals and cereal derivatives are among the main foods that contribute to Al dietary intake [18]. In 2010, González-Weller estimated the total intake of Al in the Canary Islands to be 10.171 mg/day [15].

Cd is a toxic element with a long half-life and a tendency to bioaccumulate [19]. Its presence in cultivation soils favors its transfer to and accumulation in cereals [20]. Known to compete in the body with other essential divalent cations, it affects the renal system, causing irreversible damage to the renal tubules [21,22]. In 2006, Rubio et al. [23] assessed dietary exposure to Cd in the Macaronesian archipelago of the Canary Islands, estimating the intake of Cd from cereals at 1.065 µg/day, and identifying cereals as one of the food categories contributing the most to the dietary intake of Cd. In 2012, the EFSA also identified cereals as one of the food categories that contributes most to the dietary intake of Cd in the European population [24].

Cr is mainly found in the trivalent ion form in food. Although oral Cr (III) is not particularly toxic [25], high intakes of Cr can trigger chronic kidney failure, dermatitis, bronchitis and asthma [26,27]. While cereals were found to contribute most to the dietary intake of Cr (0.087 mg/day) in the Canary Islands archipelago [28] compared to other food categories, a study by Filippini et al. [29] concluded that beverages, cereals and meat provided the highest dietary contributions of Cr in a northern Italian population.

Ni is essential for plants [30], and grains and grain-based products are considered the most important contributors to Ni exposure in the European diet, even though Ni is only regulated in drinking water and not in other food groups [9]. Individuals with hypersensitivity to Ni or with kidney disease are susceptible to damage from a high dietary intake of Ni [26].

Sr is an element that is found in food; however, there are no reported cases of food poisoning from Sr to date. Nevertheless, Sr competes with essential elements such as phosphorus [31], and recent studies in experimental animals reported hepatotoxic effects associated with Sr [32]. The total intake of Sr in the Canary Islands archipelago was estimated at 1.923 mg/day, and cereal intake was estimated at 1.276 ± 0.711 mg/kg w.w. [28].

Pb is a neurotoxic metal that accumulates in the body, causing serious damage to the central nervous system (CNS) as well as contributing to kidney disease, gastrointestinal tract disorders and Alzheimer*’*s [13]. Pb traces can be found in large quantities in food and drinking water [33,34], especially in fruits, vegetables and cereals due to the deposit of Pb particles from the atmosphere. Bread and rolls (8.5%), tea (6.2%) and tap water (6.1%) are among the food categories found to contribute to high Pb exposure in Europe [35]. While Pb intake of the Canarian population was estimated at 72.8 μg/day in 2005 [33], in 2012, mean lifetime dietary exposure in the European population was estimated at 0.68 µg/kg b.w. per day based on middle bound mean lead occurrence [35].

Food risk surveillance and food safety strategies encourage the monitoring of metal in each of the food groups consumed by different populations. The aims of the present study are to determine the levels of Al, Cd, Cr, Ni, Pb or Sr in commonly consumed cereals and cereal-based products in the Cape Verde islands, and to assess their subsequent risk.

## 2. Material and Methods

### 2.1. Samples

**Figure 1:**
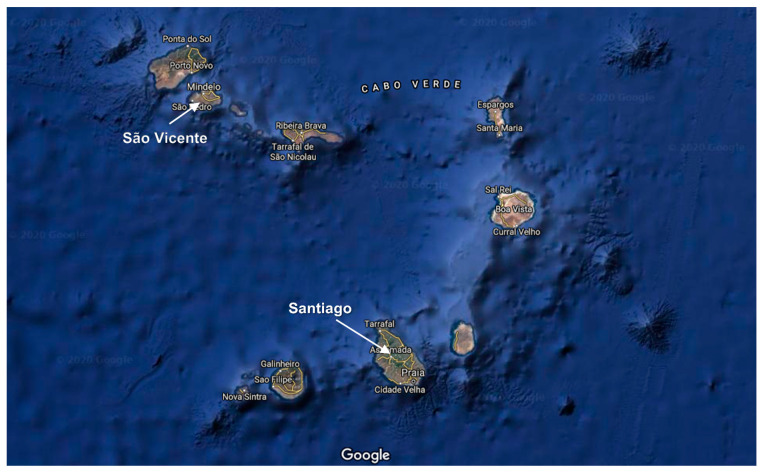
Map of the Cape Verde islands showing the sampling areas (São Vicente and Santiago) (Source: Google Maps).

A total of 126 samples of cereals (rice, corn and wheat) and cereal-based products (corn flour, wheat flour and corn gofio) (Table 2) that are marketed and consumed in Cape Verde were acquired from two different islands of the Cape Verde archipelago, specifically, Santiago and São Vicente (Figure 1). Gofio is a traditional artisan food derived from cereals, mainly corn, that is made by first roasting the cereal in its husk and then grinding it until a powder similar to flour is obtained [36,37,38].

Sampling took place from 2017 to 2019 at establishments that import and sell cereal on the Santiago and São Vicente islands. Because most of the samples were not commercialized in packages, but instead, were mainly sold by weight in local markets, it was not possible to obtain the origin of each individual sample. Nevertheless, according to Entidade Regulatora Independiente da Saúde (ERIS) from Cape Verde, the origins of the cereal samples distributed in Cape Verde are diverse (Table 2).

### 2.2. Sample Treatment

One gram of each sample was added to pressure vessels (HVT50, Anton Paar, Graz, Austria) previously washed with laboratory detergent and Milli-Q quality distilled water. Then, 4 mL 65% nitric acid (Sigma Aldrich, Darmstadt, Germany) and 2 mL hydrogen peroxide (Sigma Aldrich, Darmstadt, Germany) were added to the samples. The pressure vessels were closed and placed in a microwave oven (Multiwave Go Plus, Anton Paar, Graz, Austria) for subsequent digestion according to the conditions described in Table 3. After the samples were digested, they were transferred to 10 mL volumetric flasks and made up with Milli-Q quality distilled water. Finally, they were transferred to airtight jars with a lid for later measurement.

### 2.3. Analytical Method

The determination of metal content was conducted by Inductively Coupled Plasma Atomic Emission Spectrometry (ICP-OES) model ICAP 6300 Duo Thermo Scientific (Waltham, MA, USA), with an Auto Sampler automatic sampler (CETAX model ASX-520).

The instrumental conditions of the method comprised the following: RF power of 1150 W; gas flow (nebulizer gas flow, make up gas flow) of 0.5 L/min; injection of the sample to the 50-rpm flow pump; stabilization time of zero s [39,40]. Instrumental wavelengths (nm) of the analyzed elements were Al (167.0), Cd (226.5), Cr (267.7), Ni (231.6), Pb (220.3) and Sr (407.7).

The quantification limits of the toxic metals, calculated as ten times the standard deviation (SD) resulting from the analysis of 15 targets under reproducibility conditions [41], were: 0.012 mg/L (Al), 0.001 mg/L (Cd), 0.008 mg/L (Co), 0.003 mg/L (Ni), 0.001 mg/L (Pb) and 0.003 mg/L (Sr).

The quality control of the method (Table 4) was based on the recovery percentage obtained with reference material (SRM 1515 Apple Leaves, SRM 1548a Typical Diet, SRM 1567a Wheat Flour) under reproducible conditions. The recovery percentages obtained with the reference material were above 94% in all cases. The statistical analysis did not detect significant differences (*p* < 0.05) between the certified concentrations and the concentrations obtained.

### 2.4. Statistical Analysis

The IBM Statistics SPSS 24.0 computer software for Windows was used for statistical analysis. Two studies were conducted in order to check the significance of the differences (*p* < 0.05) in the metal contents both between cereals and derived product types and between locations. Kolmogorov-Smirnov and Shapiro-Wilk tests were used to check normality, and Levene’s test was applied to check the homogeneity of the variances based on the mean, median and trimmed mean. Data followed a non-normal distribution, and consequently, the Kruskal-Wallis nonparametric test was applied [42]. A one-way study was conducted with the fixed factor *“*Cereal type*”* and six levels of variation: *rice, corn gofio, corn flour, wheat flour, corn, wheat*. The Mann-Whitney test was also conducted (95% confidence interval) to determine significant differences in the concentrations of elements according to the cereal type or product. Another one-way study was conducted with the fixed factor *“*Location*”* and two levels of variation: *Santiago, São Vicente*. Finally, another Mann-Whitney test was used, and 166 data were analyzed with a 95% confidence interval.

### 2.5. Calculation of Dietary Intake

The assessment of dietary exposure was based on the calculation of the estimated daily intake (EDI) and the subsequent obtained percentage contribution to the reference value (TDI for Cr, Ni and Sr; TWI for Al and Cd; BMDL for Pb) of each of the metals under study (Table 1).

EDI (mg/day) = Mean consumption (kg/day) × Element concentration (mg/kg fresh weight)

Contribution (%) = [EDI/Reference value] × 100

## 3. Results and Discussion

**Figure 2:**
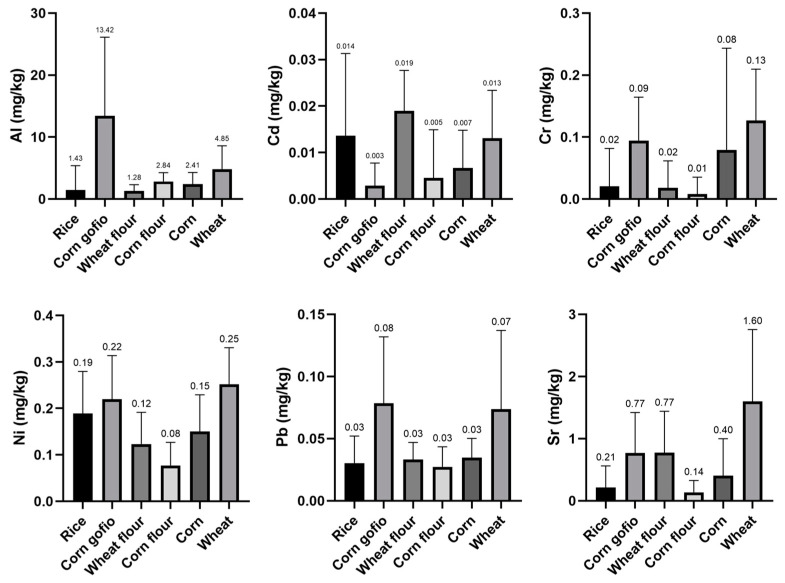
Box plot of mean trace element concentrations (mg/kg) by cereals and derived products.

Figure 2 shows box plots with the mean concentrations (mg/kg fresh weight), standard deviations (SD) and comparisons of the concentrations between the different cereals and the derived products.

Al was found in the highest concentrations in all analyzed cereal samples, most clearly in corn gofio, where it reached a mean average concentration of 13.4 ± 12.7 mg/kg fresh weight. This concentration differs significantly from the rest of the cereals (*p* < 0.05). Liu et al. [43] concluded that cereal husks contain higher concentrations of metals than the grain. Accordingly, the differences in the Al content recorded here in corn gofio may be due to the use of the whole cereal, including the husk, in the manufacture of this corn-derived product [35], which may explain the higher Al content. However, despite the toxicological considerations of this neurotoxic element, current European legislation does not include maximum levels of Al in food.

The wheat flour samples are worth mentioning, as they presented the highest levels of Sr (1.60 mg/kg fresh weight), Ni (0.25 mg/kg fresh weight) and Cr (0.13 mg/kg fresh weight). The Second French Total Diet Study (TDS) had a mean level of Sr in breakfast cereals of 0.842 mg/kg fresh weight [44]; this value was lower than the level obtained in the wheat samples of the present study. In addition, Cubadda et al. [45] reported lower Ni levels in flour and wheat (0.035 mg/kg) than those observed in this study. However, Mathebula et al. [46] observed a mean Cr level in wheat of 2.629 mg/kg fresh weight, higher than the mean level recorded in this study.

As observed for Sr, Ni and Cr, the wheat flour samples also presented the highest mean concentration of Cd (0.02 ± 0.01 mg/kg fresh weight). Tejera et al. [47] recorded mean Cd concentrations of 0.027 mg/kg fresh weight in wheat flour, values similar to those recorded in the present study. However, regarding wheat grain, Škrbić et al. [48] observed Cd levels in Serbian wheat of 2.4–252 µg/kg fresh weight, higher than those registered in the wheat analyzed here (0.01 ± 0.01 mg/kg fresh weight).

As for Pb, the highest mean level was observed in the corn gofio samples, with a mean concentration of 0.08 ± 0.05 mg/kg fresh weight. Furthermore, this concentration may indicate that Pb tends to accumulate in the husk of cereals, since in cereal-based products manufactured without the husk, the Pb levels were lower. A study conducted by Bilo et al. [49] on rice and rice husks concluded that rice husks accumulated higher concentrations of toxic metals than rice. This suggests that gofio, being a derivative produced from whole-grain cereal, including the husk, may have higher Pb levels than flours produced from dehusked cereal.

The statistical analysis showed significant differences (*p* < 0.05) in the Pb content between wheat and the rest of the samples, in the Al content between the rice and wheat samples and in the Sr and Ni content of the rice and corn samples when compared to the wheat samples.

**Figure 3:**
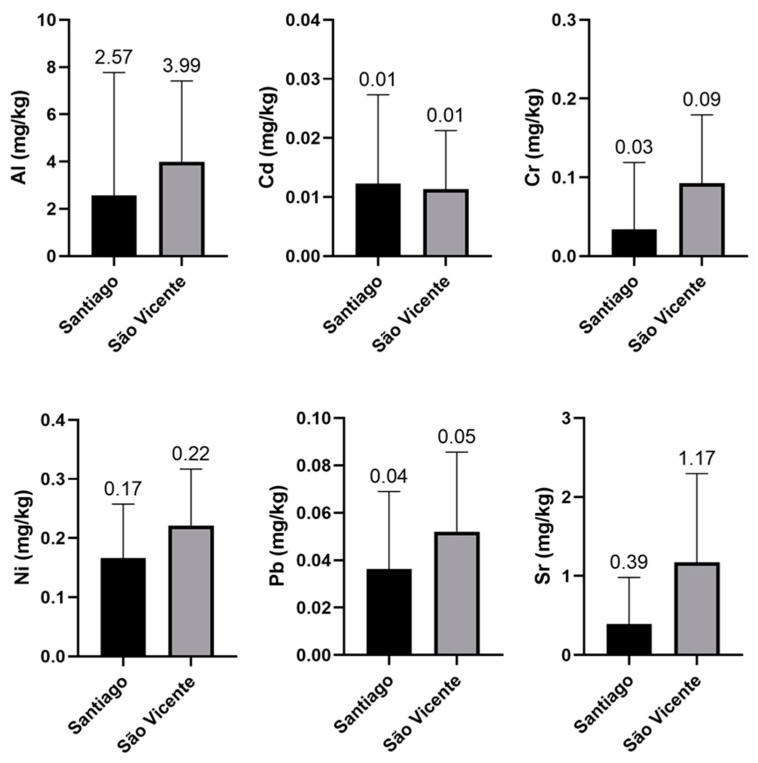
Box plot of mean trace element concentrations (mg/kg) by sampling location.

Figure 3 presents box plots with the mean concentrations (mg/kg fresh weight), standard deviations (SD) and the comparisons of the concentrations between the sampling locations. The samples from São Vicente presented the highest mean concentrations of Al, Cd, Cr, Ni, Sr and Pb. Considering that these differences may be due to multiple factors [48,50], it is suggested that in future risk-assessment studies, correlations between metal levels and the origin of the imports are calculated. Minimizing the dietary exposure of the Cape Verdean population to metals of toxicological relevance involves risk management actions, including continuous monitoring of these metals in the different food commodities upon arrival in Cape Verde, as well as importing higher-quality cereals that also have lower concentrations of Al, Cd, Cr, Ni, Sr and Pb. In addition, cereals with higher levels of metals, such as Pb and Al, should not be used for the manufacture of cereal-based products containing the husk, but rather, should be used in the manufacture of flours after being dehusked.

In Cape Verde, the cereal balance for 2002/2003 estimated a cereal consumption of 242 kg/year per person, made up of 123 kg maize (337 g/day), 67 kg rice (184 g/day) and 52 kg wheat (142 g/day). However, since there are no additional current data on the consumption habits of cereals and cereal-based products, the estimations here of the dietary exposure (Estimated Daily Intake, EDI) of the Cape Verdean population to the metals under study were performed using a mean ration of 100 g/day of each cereal and its derivatives (Table 5). The European reference limits (Table 1) were used for the evaluation of the EDI of the Cape Verde population. The TDI, TWI, and BMDL were used, along with an estimated mean average weight of an adult individual of 68.48 kg (similar to that of the Spanish population) [51].

Thus, the consumption of 100 g/day of wheat represents a contribution percentage of 13.2% to the TDI (tolerable daily intake) of Ni, i.e., 13 µg/kg bw/day. In the case of sensitive individuals or people with kidney problems, a high intake of Ni may be a dietary hazard and health risk [9]. The consumption of 100 g/day of wheat was found to provide a contribution percentage of 17.5% of the European BMDL of Pb set at 0.63 µg/kg bw/day for nephrotoxic effects [13]. This percentage may represent a relevant contribution to the total intake of Pb with the consequent risk to health. Similarly, the consumption of 100 g/day (700 g/week) of corn gofio contributes 13.7% of the TWI (tolerable weekly intake) of Al set in Europe at 1 mg/kg bw/week [11].

The Al levels detected in the corn gofio differed between the Santiago and Sào Vicente islands; in the case of Sào Vicente (39 mg Al/kg fresh weight), the consumption of 100 g/day with an Al content of 39 mg/kg fresh weight would mean an intake of 3.9 mg Al/day from this food alone, i.e., almost 39.9% of the TWI for Al.

Assuming that food risk management needs to be accompanied by a communication plan, the authors believe that the nutritional re-education campaigns and actions provided in the PERVEMAC2 Project could contribute to communicating and disseminating this knowledge to the Cape Verdean population, risk managers and policy regulators. Previous studies carried out in Cape Verde [52] have pointed to the success of involving women in health promotion because of their decision-making power; their multidimensional role in purchasing, processing and preparing food as the pillar of familial food security; and their contribution via nonformal economic activities for their families. Focus group discussions and intensive fieldwork reinforced the higher participation of residents in the informal unit and women in all stages, suggesting the practicability of health-promotion campaigns; this work also showcases the potential of the social capital of the informal settlements and the role of the woman in the family and society in Cape Verde [52].

## 4. Conclusions

In this study, the existence of significant differences in the content of elements analyzed between different cereals is confirmed, which reaffirms the need for continuous monitoring of both locally produced and imported cereals upon arrival in Cape Verde as risk management and minimization strategies, while also continuing to monitor the population’s total dietary exposure to toxic metals. Furthermore, cereals with higher levels of metals such as Pb and Al should not be used with the husk for the manufacture of cereal-based products, but rather, should be used in the manufacture of flours only after removing the husk. In the case of Al, it would be advisable for the food safety authorities to set a maximum limit for this element in cereals and cereal-based products, thus allowing quality control and minimizing the population’s exposure to this neurotoxic element. The evaluation of dietary exposure to the toxic metals studied here in cereals and their cereal-based products should undoubtedly be complemented with future studies targeting other groups of basic foods in the diet of the Cape Verde population.

## Figures and Tables

**Table 1:** Reference intakes of the analyzed elements.

Element	Parameter	Guideline Value	References
Cr (III)	TDI	0.3 mg/kg bw/day	[8]
Ni		13 µg/kg bw/day	[9]
Sr		0.13 mg/kg bw/day	[10]
Al	TWI	1 mg/kg bw/week	[11]
Cd		2.5 µg/kg bw/week	[12]
Pb	BMDL	0.63 ^1^ µg/kg bw/day 1.50 ^2^ µg/kg bw/day	[13]

TDI, tolerable daily intake; TWI, tolerable weekly intake; BMDL, benchmark dose level; bw, body weight; Nephrotoxicity ^1^ and Cardiovascular effects ^2^.

**Table 2:** Analyzed cereal and derived product samples.

Type	No. Samples	Sampling Location	Origin
Rice	56	Santiago	Brazil, Vietnam, Thailand, Japan, USA (California), Cape Verde (Mindelo), Pakistan
	5	São Vicente	
Corn gofio	6	Santiago	Unknown
	1	São Vicente	
Corn flour	10	Santiago	Portugal, The Netherlands
	1	São Vicente	
Wheat flour	17	Santiago	Portugal, France
	2	São Vicente	
Corn	13	Santiago	Argentina, France, Russia, South America
	2	São Vicente	
Wheat	2	Santiago	Russia, France, Cape Verde (Mindelo), Spain
	11	São Vicente	

**Table 3:** Microwave digestion process instrumental conditions.

No.	Ramp (min)	Temperature (°C)	Time (min)
1	15	50	5
2	5	60	4
3	5	70	3
4	3	90	2
5	20	180	10

Microwave processing power: 850 W; Limit temperature: 200 °C; Cooling temperature: 50 °C.

**Table 4:** Recovery study results and reference materials used.

Metal	Material	Concentration Found (mg/kg)	Certified Concentration (mg/kg)	R (%)
Al	SRM 1515 Apple Leaves	286 ± 9	285.1 ± 26	99.7
Sr		25.0 ± 2.0	24.6 ± 4.0	98.3
Cr		0.29 ± 0.03	0.30 ± 0.00	97.8
Ni	SRM 1548a Typical Diet	0.37 ± 0.02	0.38 ± 0.04	102.3
Pb		0.044 ± 0.000	0.044 ± 0.013	98.9
Cd	SRM 1567a Wheat Flour	0.026 ± 0.002	0.026 ± 0.008	98.4

**Table 5:** Metal dietary intake assessment and evaluation.

Element	EDI (mg/day)	Contribution	EDI (mg/day)	Contribution	EDI (mg/day)	Contribution	EDI (mg/day)	Contribution	EDI (mg/day)	Contribution	EDI (mg/day)	Contribution
	Rice		Corn		Corn Flour		Wheat Flour		Corn Gofio		Wheat	
Cr	0.002	0.01% TDI	0.008	0.04%TDI	0.001	0.005%TDI	0.002	0.01%TDI	0.009	0.04%TDI	0.01	0.05%TDI
Ni	0.02	10.00% TDI	0.02	7.89%TDI	0.008	4.21%TDI	0.01	6.32%TDI	0.02	11.6%TDI	0.03	13.2%TDI
Sr	0.02	0.24% TDI	0.04	0.45%TDI	0.01	0.16%TDI	0.08	0.87%TDI	0.08	0.87%TDI	0.16	1.80%TDI
Al	0.14	1.46% TWI	0.24	2.46%TWI	0.12	1.20% TWI	0.27	2.80% TWI	1.34	13.7% TWI	0.49	4.96% TWI
Cd	0.001	4.09% TWI	0.0007	2.86%TWI	0.0005	2.04% TWI	0.002	8.18% TWI	0.0003	1.23% TWI	0.001	4.09% TWI
Pb	0.003	7.50% BMDL for Nephrotoxicity	0.003	7.50% BMDL for Nephrotoxicity	0.003	7.50% BMDL for Nephrotoxicity	0.003	7.50% BMDL for Nephrotoxicity	0.008	20.0% BMDL for Nephrotoxicity	0.007	17.5%BMDL for Nephrotoxicity
		3.00% BMDL for Cardiovascular Effects		3.00% BMDL for Cardiovascular Effects		3.00% BMDL for Cardiovascular Effects		3.00% BMDL for Cardiovascular Effects		8% BMDL for Cardiovascular Effects		7% BMDL for Cardiovascular Effects

Estimated daily intake (mg/day) when consuming 100 g/day; Percentage of contribution (%) to the Reference Intake (Table 1) when consuming 100 g/day. Considering a mean average weight of an adult of 68.48 kg [50].

## Data Availability

The datasets generated during the current study are not publicly available but are available from the corresponding author on reasonable request.
